# Prognostic Factors and Visual Outcomes of Pyogenic Liver Abscess-Related Endogenous *Klebsiella pneumoniae* Endophthalmitis: *A 20-year retrospective review*

**DOI:** 10.1038/s41598-018-37643-y

**Published:** 2019-01-31

**Authors:** Yi-Hua Chen, Ya-Han Li, Yu-Jr Lin, Yen-Po Chen, Nan-Kai Wang, An-Ning Chao, Laura Liu, Wei-Chi Wu, Chi-Chun Lai, Tun-Lu Chen, Kuan-Jen Chen

**Affiliations:** 1 Department of Ophthalmology, Chang Gung Memorial Hospital, Chang Gung University College of Medicine, Taoyuan, Taiwan; 2 Department of Ophthalmology, Mackay Memorial Hospital, Hsinchu, Taiwan; 3 Research Services Center for Health Information, Chang Gung University, Taoyuan, Taiwan; 4 Department of Ophthalmology, Edward S. Harkness Eye Institute, Columbia University, New York, NY 10032 United States

## Abstract

Endogenous *K. pneumoniae* endophthalmitis (EKE) has a higher incidence among East Asians, and the most common infectious source of EKE is pyogenic liver abscess (PLA). We investigate the risk factors for poor visual outcomes in patients with PLA-related EKE. The retrospective medical records of 104 patients (120 eyes) diagnosed with PLA-related EKE between 1996 and 2015. In univariate logistic regression analysis, the risk factors for poor visual outcomes were initial visual acuity (VA) worse than counting fingers (CF) (*p* < 0.001), eye pain (*p* = 0.013), hypopyon (*p* = 0.003), ocular hypertension (*p* = 0.003), *p*ositive intraocular fluids cultures (*p* < 0.001), subretinal abscess (*p* = 0.025), unilateral involvement (*p* = 0.017), delayed ophthalmologic visit (*p* = 0.022), initially presented with ocular symptoms ahead of systemic symptoms (*p* < 0.001), and corneal edema (*p* < 0.001). Intravitreal dexamethasone reduced the requirement of enucleation or evisceration (*p* = 0.01). The multivariate logistic regression revealed that poor initial VA worse than CF (*p* = 0.004) and initially presented with ocular symptoms ahead of systemic symptoms (*p* = 0.007) were the significant independent factors for poor visual outcomes. Early diagnosis and prompt treatment may salvage useful vision in some eyes.

## Introduction

Endogenous endophthalmitis is a rare but potentially sight-threatening disease. It is caused by the transport of pathogens from other infectious focuses to the internal eyes through the blood–ocular barrier. Endogenous endophthalmitis in East Asian and Western countries has shown distinct causative organisms^1,2^. Gram-positive bacteria and *Candida* were the most common pathogens among the Western countries with more favorable visual outcomes, and gram-negative bacteria were the main causative organisms in East Asian countries^2–5^. In East Asia, *Klebsiella pneumoniae* is the predominant pathogen, and its increasing incidence has been reported worldwide^2,4,5^.

The incidence of endogenous *K. pneumoniae* endophthalmitis (EKE) among patients with systemic *K. pneumoniae* infection ranged from 3.8% to 11% in Asian countries^6–8^. Furthermore, it has a higher incidence among East Asians^9–11^. The most common infectious source of EKE is pyogenic liver abscess (PLA)^2,6,8,12^. Moreover, salvageable vision is limited to eyes that receive early treatment^7,8,13^.

This purpose of study is to investigate the prognostic factors for poor visual outcomes, including subjective symptoms, clinical features at presentation, laboratory data, and treatments, in patients who received a diagnosis of PLA-related EKE in a 20-year period at a tertiary referral center in Northern Taiwan.

## Results

A total of 140 patients developed EKE between January 1996 and December 2015. Among them, five patients had co-infections with liver abscess and other infectious focuses, such as lung, prostate, renal abscesses and limb cellulitis. However, 31 patients had infections located outside of the liver, and were therefore excluded from our study. In total, 120 eyes of 104 patients with PLA-related EKE were evaluated in our study. Table 1 demonstrates patients’ characteristics, systemic illnesses, ocular features at presentation, and final visual outcomes, and Table 2 shows the prognostic factors for poor visual outcomes.

**Table 1:** Demographics, Systemic illness, Clinical features, and Outcomes of Patients with Liver abscess related Endophthalmitis.

Demographics	Eye or Patient No. (%)	Clinical features	Eye No. (%)
Patient number	104	Presenting visual acuity	
Eyes effected	120	Worse than CF	76(64.3%)
OD	55(45.8%)	CF or better	44(35.7%)
OS	65(54.2%)	At presentation	
Mean age, years (±SD)	58.3 ± 11.7	EE previous than PLA	62(52.1%)
Sex		PLA previous than EE	57(47.9%)
Male	68(65.4%)	NA	1 (0.8%)
Female	36(34.6%)	Elevated IOP	
Systemic illness		Yes	46(38.3%)
Diabetes	71(68.3%)	No	74(61.7%)
Hypertension	33(31.7%)	Hypopyon	
Liver abscesses		Yes	49(40.8%)
Single	77(74.0%)	No	71(59.2%)
Multiple	18(17.3%)	Laterality	
NA	9 (8.7%)	Unilateral	88(73.3%)
Location of abscesses		Bilateral	32(26.7%)
Right lobe	63(60.6%)	Vitrectomy	
Left lobe	13(12.5%)	Early PPV for infection control	26(21.7%)
Both lobes	10(9.6%)	Late PPV for sequelae	9(7.5%)
NA	18(17.3%)	Outcomes	
Maximal diameter of abscesses, cm (±SD)	5.6 ± 2.4	Final visual acuity	
		Good: CF or better	35(29.2%)
		Poor: worse than CF	85(70.8%)
		Evisceration/Enucleation	34(28.3%)
		Phthisis	34(28.3%)

Note: CF, counting fingers; SD, standard deviation; EE, endogenous endophthalmitis; NA, not available; PLA, pyogenic liver abscess; PPV, pars plana vitrectomy; VA, visual acuity.

**Table 2:** Prognosis factors for poor visual outcome.

Factors		Final visual outcome		Univariate^a^		Multivariate^b^	
				OR (95% CI)	*P value*	OR (95% CI)	*P value*
		CF or better	Worse than CF				
Patient characteristics	No. of eyes	No. of eyes (%)/ Mean ± SD					
Age, years^c^		55.2 ± 10.9	56.9 ± 12.1		0.066		
Length of hospital stay, days^c^		24.2 ± 10.1	26.7 ± 11.8		0.283		
Sex^d^							
Male	79	28(35.4%)	51(64.6%)	1			
Female	41	7(17.1%)	34(82.9%)	2.6(1.0–6.8)	0.060		
Laterality^e^							
Unilateral	88	21(23.9%)	67(76.1%)	2.8(1.2–6.6)			
Bilateral	32	15(46.9%)	17(53.1%)	1	0.017*		
Diabetes^d^	Y = 82	23(28.0%)	59(72.0%)	1.2(0.5–2.7)			
	N = 38	12(31.6%)	26(68.4%)	1	0.693		
Hypertension^d^	Y = 46	11(23.9%)	35(76.1%)	1.5(0.7–3.5)			
	N = 74	24(32.4%)	50(67.6%)	1	0.314		
Liver abscesses^d^							
Single	89	28(31.5%)	61(68.5%)	1			
Multiple	20	5(25.0%)	15(75.0%)	1.4(0.5–4.1)	0.564		
Location of abscesses^e^							
In right or left lobe	86	28(32.6%)	58(67.4%)	1			
In both lobes	10	1(10%)	9(90%)	4.3(0.5–36.0)	0.273		
Maximal diameter of abscesses, cm^c^		5.3 ± 2.4	5.8 ± 2.5	1.1(0.9–1.3)	0.315		
**Presenting clinical features**							
Eye pain^d^	Y = 79	18(22.8%)	61(77.2%)	2.8(1.2–6.3)			
	N = 40	18(45.0%)	22(55.0%)	1	0.013*		
Fever^e^	Y = 104	32(30.8%)	72(69.2%)	1			
	N = 15	3(20%)	12(80%)	1.8(0.5–6.7)	0.548		
Chillness^d^	Y = 80	26(32.5%)	54(67.5%)	1			
	N = 38	9(23.7%)	29(76.3%)	1.6(0.6–3.7)	0.327		
Abdominal pain^d^	Y = 57	20(35.1%)	37(64.9%)	1			
	N = 62	15(21.2%)	47(75.8%)	1.7(0.8–3.7)	0.193		
**At presentation** ^d^							
EE previous than PLA	62	8(12.9%)	54(87.1%)	6.1(2.5–15.0)			
PLA previous than EE	57	27(47.4%)	30(52.6%)	1	<0.001*	5.1(1.6–16.6)	0.007*
Times of ocular symptoms to ophthalmologist consult, days^c^		2.1 ± 1.5	2.9 ± 1.8	1.4(1.1–1.9)	0.022*		
**Ocular and laboratory examination**							
Initial VA worse than CF^d^	Y = 76	10(13.2%)	66(86.8%)	8.7(3.6–21.2)			
	N = 44	25(56.8%)	19(43.2%)	1	<0.001*	4.6(1.6–13.4)	0.004*
Ocular hypertension^d^	Y = 46	4(8.7%)	42(91.3%)	7.6(2.5–23.3)			
	N = 74	31(41.9%)	43(58.1%)	1	<0.001*		
IOP, mmHg^c^		16.5 ± 9.9	26.3 ± 11.7	1.1(1.0–1.2)	0.003*		
Corneal edema^d^	Y = 82	16(20%)	66(80%)	6.4(2.3–18.0)			
	N = 36	19(52.8%)	17(47.2%)	1	<0.001*		
Hypopyon^d^	Y = 49	7(14.3%)	42(85.7%)	3.9(1.5–9.9)			
	N = 71	28(39.4%)	43(60.6%)	1	0.003*		
Subretinal abscess^e^	Y = 113	30(26.5%)	83(73.5%)	6.9(1.3–37.6)	0.025*		
	N = 7	5(71.4%)	2(28.6%)	1			
WBC count (x 10^9^/L)^c^		17.3 ± 14.5	14.2 ± 6.5		0.244		
Thrombocytopenia^d^	Y = 63	26(41.3%)	37(58.7%)	1			
(<100,000/mm^3^)	N = 49	8(16.3%)	41(83.7%)	3.6(1.5–8.9)	0.006*		
AST^c^		118.9 ± 127.8	58.4 ± 51.6		0.076		
ALT^c^		109.7 ± 111.2	78.3 ± 68.0		0.183		
Alk-P^c^		195.7 ± 115.5	209.8 ± 112.4		0.671		
HbA1c in DM patients^c^		10.0 ± 3.2	8.8 ± 3.0		0.245		
Blood culture^d^	Y = 84	25(29.8%)	59(70.2%)	1			
	N = 36	10(27.8%)	26(72.2%)	1.1(0.5–2.6)	0.826		
Aqueous or vitreous culture^d^	Y = 40	2(5.0%)	38(95.0%)	13.3(3.0–59.2)			
	N = 80	33(41.2%)	47(58.8%)	1	<0.001*	4.9(0.9–26.4)	0.059
**Treatment**							
Abscess drainage^d^	Y = 68	24(35.3%)	44(64.7%)	1			
	N = 52	11(21.2%)	41(78.8%)	2.0(0.9–4.7)	0.091		
IVI of antibiotics^d^							
With dexamethasone	34	14(41.2%)	20(58.8%)	1			
Without dexamethasone	86	21(24.4%)	65(75.6%)	2.2(0.9–5.0)	0.069		
Early TPPV^d^	Y = 26	5(19.2%)	21(80.8%)	2.0(0.7–5.7)			
	N = 94	30(31.9%)	64(68.1%)	1	0.208		

Note; CF, counting fingers; CI, confidence interval; IOP, intraocular pressure; IVI, intravitreal injection; TPPV, trans pars plana vitrectomy; VA, visual acuity; WBC, white blood cell; EE, endogenous endophthalmitis; PLA, pyogenic liver abscess; AST, aspartate aminotransferase; ALT, alanine aminotransferase; Alk-P, alkaline phosphatase; *statistical significant ^a^univariate logistic regression; ^b^Binary logistic regression; ^c^Student’s t test; ^d^Pearson’s chi square test; Fisher’s Exact test.

### Patients’ characteristics

The mean age of our study group was 58.3 ± 11.7 years (33–87 years). The mean days of hospitalization to complete treatment course were 25.7 ± 11.2 days (2–53 days). Men were nearly two times (68 of 104 patients, 65.4%) more likely to have the diseases than women (36 of 104 patients, 34.6%); however, they did not exhibit substantial differences in their visual outcomes. Most patients had unilateral involvement (88 of 104 patients, 84.6%); however, both eyes had a similar risk of endogenous endophthalmitis (right eye, 55 of 120 eyes, 45.8%; left eye, 65 of 120 eyes, 54.2%). Furthermore, 71 of 104 patients (68.3%) had diabetes mellitus, which has been considered as a common problem in immunocompromised patients. In addition, 11 (68.8%) of 16 patients with bilateral endophthalmitis had diabetes mellitus.

Single abscess (89 of 109 eyes, 81.7%; 77 of 104 patients, 74%) was more commonly identified than multiple abscesses (20 of 109 eyes, 18.3%; 18 of 104 patients, 17.3%) in imaging studies. The mean maximal diameter of the abscess was 5.6 ± 2.4 cm (0.7–12 cm). Abscesses located over the right lobe of the liver were predominant (73 of 96 eyes, 76.0%; 63 of 104 patients, 60.6%); however, the location, number, or size of liver abscesses were not associated with poor visual outcomes. In addition, 56 patients (53.3%) who received catheter drainage placement did not exhibit differences in visual outcomes compared with those without this placement.

### Clinical features at presentation

Common ocular complaints at the time of presenting to our hospital included eye pain (79 of 119 eyes, 66.4%) and decreased VA (114 of 120 eyes, 95.0%). Fever (104 of 119 eyes, 87.4%; 89 of 104 patients, 85.6%) and chillness (80 of 118 eyes, 67.8%; 67 of 104 patients, 64.4%) were also identified. The symptoms, such as abdominal pain, of liver abscess were included. Forty-nine patients (57 of 119 eyes, 47.9%) were detected as having abdominal pain at presentation.

Sixty-two of 119 eyes (52.1%) were confirmed to have endogenous endophthalmitis before receiving a diagnosis of PLA. Poor visual outcomes (odds ratio [OR], 6.1; 95% confidence interval [CI], 2.5–15.0; *p* < 0.001) were observed in this group. Patients who initially presented with ocular complaints had subsequent poorer visual outcomes compared with those who presented with abdominal or general complaints. The early detection of endophthalmitis in patients who present with ocular complaints is crucial. The mean duration from ocular symptom presentation to receiving medical treatment was 2.6 ± 1.7 days (1–21 days), and it was significantly associated with poor visual outcomes in delayed consultation (OR, 1.4; 95% CI, 1.0–1.9; *p* = 0.022). The mean delayed days in the final VA worse than CF group was 2.9 ± 1.8 days, which was almost 1 day slower than the better final vision group who seek medical help (2.1 ± 1.5 days). Eye pain (OR, 2.8; 95% CI, 1.2–6.3; *p* = 0.013) was a poor prognostic factor, whereas fever, chillness, and abdominal pain were not associated with visual outcomes.

### Ocular examinations

Seventy-six eyes (63.3%) had an initial VA worse than CF, including no light perception (NLP) in 22 eyes (18.3%), light perception (LP) in 28 eyes (23.3%), and hand motion (HM) in 26 eyes (21.7%). The other 44 eyes (36.7%) had an initial VA equal or better than CF, including CF in 25 eyes (20.8%), VA between 2/200 to 20/200 in 10 eyes (8.3%), and VA between 20/100 to 20/20 in 9 eyes (7.5%). An initial VA worse than CF was prognostic factor for poor visual outcomes (OR, 8.7; 95% CI, 3.6–21.2; *p* < 0.001). Only 35 of 120 eyes (29.2%) had a final VA equal or better than CF.

Elevated IOP of over 21 mmHg was defined as ocular hypertension. 46 of 120 eyes (38.3%) had ocular hypertension. The mean IOP in the poor final VA group was 26.3 ± 11.7 mmHg, which was significantly higher than that of the favorable final VA group (16.5 ± 9.9 mmHg; OR, 1.1; 95% CI, 1.0–1.2; *p* = 0.003). Furthermore, one third (36/118) of the eyes exhibited clear cornea at presentation, whereas 78% (64/82) eyes with corneal edema had a significantly poor final VA (OR, 6.4; 95% CI, 2.3–18.0; *p* < 0.001). The presence of a hypopyon was identified as a risk factor for poor VA outcomes (OR, 3.9; 95% CI, 1.5–9.9; *p* = 0.003). The mean height of the hypopyon was 1.8 ± 1.4 mm in 49 of 120 eyes; however, the height of hypopyon did not affect final visual outcomes in this study. Subretinal abscess was identified at presentation by fundoscopic and ocular sonographic examination or during the TPPV. 113 eyes (94.2%) had subretinal abscess, but 73.5% (83/113) of them presented with poor finial VA. Eyes having subretinal abscess had a prognostic factor for poor visual outcomes (OR, 6.9; 95% CI, 1.3–37.6; *p* = 0.0252).

### Laboratory examinations

Leukocytosis (>12,000/mm^3^) was observed in 55% (55/100) of patients, and only one patient had leukopenia (<3,500/mm^3^). The better and worse final VA groups did not differ in their white blood cell count. Moreover, more than half of the eyes had underlying moderate thrombocytopenia (<100,000/mm^3^; 63/120 eyes in 51 patients, 52.5%), and six eyes had thrombocytosis (>450,000/mm^3^; 6/120 eyes in 6 patients, 5%). Among the thrombocytopenia group, 14 patients had bilateral endophthalmitis, accounting for 87.5% of 16 patients with bilateral endophthalmitis. Moderate thrombocytosis is considered to be a risk factor for poor visual outcomes (OR, 3.6; 95% CI, 1.5–8.9; *p* = 0.006) in univariate analysis and increased probability of bilateral endophthalmitis.

Blood cultures were routinely performed on all patients who presented with infectious symptoms at our emergent department or wards. Blood cultures were positive for *K. pneumoniae* in 74 patients (84 of 120 eyes, 70%), whereas *K. pneumoniae* growth was not observed in 30 patients. Cultures from the aqueous humor or vitreous or from both sites were performed in our patients before intravitreal antibiotic administration. Cultures in one third (40/120) of the eyes were positive for *K. pneumoniae* and was identified as a risk factor for poor visual outcomes (OR, 13.3; 95% CI, 3.0–59.2; *p* < 0.001); however, positive *K. pneumoniae* blood cultures were not associated with final visual prognosis.

### Treatments

Intravitreal antibiotics were administered to all eyes in this study. All 40 eyes with positive *K. pneumoniae* from aqueous or vitreous culture results revealed sensitive to ceftazidime or amikacin. There was no statistical difference between groups of eyes who were treated with ceftazidime and amikacin. Additionally, thirty-four eyes (28.3%) received intravitreal dexamethasone. It showed marginally significant differences in their visual outcomes (*p* = 0.069) between groups with and without intravitreal dexamethasone. However, during follow-up, 30 eyes required enucleation or evisceration in the non-dexamethasone treatment group, which was significantly higher than that (4 eyes) in the dexamethasone treatment group (OR, 4.0; 95% CI, 1.3–12.5; *p* = 0.01). Furthermore, 26 eyes (26/120, 21.7%) received early TPPV for infection control. No significant differences in visual outcomes were observed between the eyes with and without early TPPV (OR, 2.0; 95% CI, 0.7–5.7; *p* = 0.208). No surgical drainage was performed in eyes with subretinal abscess during the TPPV. At the end of TPPV, gas or silicone oil was used for retina tamponade. Because of small number of eyes, there was no statistical difference in final visual outcomes between gas and silicone oil tamponade groups. However, patients receiving early TPPV marginally significantly improved VA (OR, 2.5; 95% CI, 1.0–6.1; *p* = 0.055) from initial presentation to last follow-up.

### Multivariate analysis for poor visual outcomes

In final visual outcomes, 72 eyes had VA with NLP, including 34 eviscerated or enucleated eyes, and eleven eyes had VA with LP. In addition, two, five, and nine eyes had a HM, CF, and VA of 2/200 to 20/200, respectively. Furthermore, 11 eyes regained a favorable VA (20/100 to 10/20), and 10 eyes regained an excellent VA (20/30 to 20/20). However, despite aggressive treatment, 85 of 120 eyes had a final VA worse than CF. During follow-up, 34 eyes were eviscerated or enucleated (28.3%) and 34 sightless eyes became phthisical (28.3%).

The significant poor prognostic factors (*p* < 0.05) for visual outcomes obtained from the univariate analysis (Table 2) were included in the multivariate analysis. Poor initial VA (OR, 4.6; 95% CI, 1.6–13.4; *p* = 0.004), positive *K. pneumoniae* aqueous or/and vitreous humor culture (OR, 4.9; 95% CI, 0.9–26.4; *p* = 0.059), and the diagnosis of endogenous endophthalmitis before PLA (OR, 5.1; 95% CI, 1.6–26.6; *p* = 0.007) were important risk factors for poor visual outcomes compared with other significant factors of the univariate analysis.

## Discussion

PLA is a rare but potentially fatal condition, with an incidence of 20 per 100,000 hospital admissions in a Western population^14^. The right lobe of the liver is the most likely site of infection probably because of an unequal distribution of the superior and inferior mesenteric vein contents within the portal venous distribution^15^. Several studies in Taiwan, Korea, and Singapore have revealed that liver abscess is the most common source (25%–52.7%) of infection for endogenous endophthalmitis^16–19^. A population-based database study in Taiwan that included 12,727 patients revealed that endophthalmitis was diagnosed in 0.84% of patients with PLA^20^. Park *et al*.^11^ reported that an abscess in the right superior segment is significantly associated with PLA-related endogenous endophthalmitis. In our study, the abscesses were commonly located in the right lobe, which is consistent with previous studies. However, the location, size, or the number of abscesses were not significantly associated with final visual outcomes. The size and number of abscesses in patients were recorded during the first abdominal ultrasound or computed tomography scan. Being a tertiary referral center, visiting patients might undergo primary treatment at other hospitals. Therefore, the size and number of abscesses may vary from that reported at initial presentation, and therefore introduce bias in data recording. Although catheter drainage placement in patients with liver abscess can result in earlier clinical improvement and 50% decrease in the abscess cavity volume^21^, the presence or absence of catheter drainage placement did not affect the final visual outcomes of the study patients.

The prevalence of endogenous endophthalmitis in patients with PLA has been reported to be 0.84%–6.9%^7,13,20^. Although endogenous endophthalmitis is rare, the outcomes are usually dismal. It is one of the most severe septic complications of PLA. In our study, only 21 eyes (17.5%) regained an acceptable VA of more than 20/100 despite early treatment, including intravitreal antibiotic administration or TPPV. Yang *et al*.^8^ revealed that two eyes regained an acceptable VA of more than 6/60 after early intravitreal corticosteroid administration. Jackson *et al*.^22^ observed that a few eyes affected with endogenous endophthalmitis that received intravitreal steroids were more likely to salvage useful vision. Whereas several publications revealed no better visual results of the cases had additional administration of corticosteroids^23–25^.

Furthermore, our previous retrospective study between January 1996 and April 2013 on EKE achieved improved visual outcomes in the intravitreal dexamethasone administration group in a multivariate analysis^26^. In the present study, a final VA better than CF was observed in up to 41.2% of eyes in the intravitreal dexamethasone administration group compared with that in only 24.4% of eyes in the intravitreal antibiotic administration without dexamethasone group. However, a simple univariate analysis showed marginally significantly favorable visual outcome results (*p* = 0.069). In the group of VA worse than CF at presentation, the number and percentage of eyes with light perception or no light perception was more than those of eyes in our previous study^26^. These eyes with light perception or no light perception were treated with intravitreal dexamethasone, but they still couldn’t achieve favorable visual outcomes because of severe damages of intraocular tissue. It might explain that our result couldn’t get statistically significantly improved visual outcomes in the intravitreal dexamethasone group. Compared with our previous retrospective study^26^, the current study only focused on the most common infectious source (PLA) associated with EKE. All *K. pneumoniae* isolated from body fluids showed sensitive to ceftazidime and amikacin. There was no extended-spectrum β-lactamases-producing *K. pneumoniae* isolated. Since we started to give dexamethasone as an empirical intravitreal drug recently, the percentage of receiving intravitreal dexamethasone in both poor and better initial VA groups had been increasing. Although intravitreal dexamethasone showed marginally significantly favorable visual outcome results, necessary evisceration or enucleation occurred less often in the patients who received intravitreal dexamethasone injections (p = 0.01). Recently, Chen *et al*.^27^ demonstrated that evisceration or enucleation prevention can be achieved in endogenous bacterial panophthalmitis with no LP and scleral abscess through the administration of multiple intravitreal and periocular antibiotics and dexamethasone. Future randomized control trials may be required to verify the effectiveness of intravitreal dexamethasone.

Early TPPV was provided for infection control and vision salvage in 26 eyes (21.7%); however, early TPPV did not successfully improve visual outcomes, and half of the eyes required evisceration or enucleation during follow-up. By contrast, some studies have demonstrated improved visual outcomes or successful vision salvage after early TPPV^16,17^. We believe that the poor response might be associated with *K. pneumoniae* infection. *K. pneumoniae* has been implicated in the rapid and irreversible destruction of the retinal photoreceptor as early as 48 hours after infection in an experimental rabbit model^28^. Because of its virulent effects on eyes, only 44 eyes (36.7%) had an initial VA better than CF in this study. *K. pneumoniae* has recently become an emerging pathogen in liver abscess in Western countries and remains the most common pathogen of liver abscess in East Asian countries, including Taiwan, for the decades^10,29^. In the present study, 84 patients (80.8%) with PLA had positive *K. pneumoniae* blood cultures, which was higher than that (50%) reported by Lau *et al*.^30^. Several studies have reported *K. pneumoniae* to be an important risk factor for PLA-related EKE^8,11^. Regarding the origin of positive cultures, only cultures of *K. pneumoniae* obtained from vitreous and aqueous humor were associated with poor visual outcomes (*p* < 0.001).

Thrombocytopenia is observed among patients with liver abscess, particularly in elderly and female patients. Yoon *et al*.^31^ concluded that clinicians must be aware of the potential metastatic infections in patients with *K. pneumoniae*-related PLA, particularly if they have diabetes mellitus and thrombocytopenia (<80,000/mm^3^). Lin *et al*.^32^ identified six independent predictors of the severe complications of *K. pneumoniae*-related liver abscess, including thrombocytopenia (<100,000/mm^3^), alkaline phosphatase >300 U/L, gas formation in the abscess, APACHE III score >40, cefazolin use, and delayed drainage. Notably, thrombocytopenia, alkaline phosphatase, and the presence or absence of drainage placement did not affect the final visual outcomes of our study; however, moderate thrombocytopenia was observed in 87.5% of patients with bilateral endogenous endophthalmitis. This result may be attributed to the incomplete data availability or low association between liver abscess-related endogenous endophthalmitis and thrombocytopenia.

The multivariate logistic regression revealed that poor initial VA worse than CF (*p* = 0.004) and initially presented with ocular symptoms ahead of systemic symptoms (*p* = 0.007) were the significant independent factors for poor visual outcomes. However, this study also has several limitations because of its retrospective nature. Some parts of the detailed data may be missing or recall bias may exist. Furthermore, we were unable to achieve a head-to-head comparison due to the lack of EKE treatment guidelines and the rarity of this disease.

In conclusion, PLA-related EKE typically leads to poor visual prognosis, particularly in initial VA worse than CF and initially presented with ocular symptoms ahead of systemic symptoms. Early diagnosis and prompt treatment may salvage useful vision in some eyes. According to our study and the high incidence of EKE in Asia, we recommend that patients who are diagnosed or suspected with *K. pneumoniae* related pyogenic liver abscess undergo routine ophthalmic examination even if there are no ocular symptoms.

## Methods

The present study is a retrospective, consecutive, comparative, and interventional case series of patients who received a diagnosis of PLA-related EKE between January 1996 and December 2015 at a tertiary referral center in Northern Taiwan. The Institutional Review Board of Chang Gung Memorial Hospital in Taoyuan, Taiwan, approved the study protocol and waived the requirement of written informed consent. All methods were performed in accordance with the relevant guidelines and regulations. The diagnoses of PLA and endogenous endophthalmitis were confirmed on the basis of the clinical characteristics and ocular, laboratory, and imaging examination results (abdominal ultrasound or computed tomography). The inclusion criteria were as follows: (1) patients who received a diagnosis of PLA and endogenous endophthalmitis at presentation or during admission, (2) those having PLA as the primary infectious source and at least one positive *K. pneumonia* culture from their body fluid (blood, liver abscess, and aqueous and vitreous humor) samples, (3) those followed up for at least 3 months, and (4) those who did not undergo ocular surgery within 6 weeks and had no history of ocular trauma. The exclusion criteria were as follows: (1) patients in which the primary infectious source was not identified or was outside the liver and (2) those having an onset interval of more than 14 days between PLA identification and EKE.

All patients, suspected with infection, have received empirical systemic antibiotics before PLA was diagnosed. After PLA was diagnosis, patients received intravenous ceftriaxone alone in patients without sepsis or combined with additional antibiotics in patients with sepsis. Ophthalmological consultation was provided during patients’ hospital stay, upon physician’s request, or during patients’ visit to the emergency or ophthalmology department due to ocular symptoms or suspicion of EKE. Immediately after endogenous endophthalmitis diagnosis, vancomycin (1.0 mg/0.1 mL) and ceftazidime (2.25 mg/0.1 mL) or amikacin (0.4 mg/0.1 mL) were administered intravitreally. Some patients also received intravitreal dexamethasone (0.4 mg/0.1 mL) along with antibiotics initially or during retreatment. Intravitreal antibiotic administration with or without dexamethasone was repeated every 48–72 hours, according to the severity and clinical response assessments of the retina specialists. Patients who ever received dexamethasone in the first two injections were classified as the group of treatment with dexamethasone. Trans pars plana vitrectomy (TPPV) was performed in patients with uncontrollable infection and poor clinical responses to intravitreal and systemic antibiotic administration such as deteriorating VA, increasing hypopyon or vitreous opacity. Evisceration or enucleation was performed in patients with eyeball perforation or painful sightless eyes despite aggressive treatments.

Data on the demographic and clinical features at presentation, medical history, laboratory data, and treatments of patients with EKE were obtained. Ophthalmic examinations included initial and final visual acuity (VA) assessments, slit-lamp biomicroscope, intraocular pressure (IOP) measurement, and indirect ophthalmoscopy or ultrasonography. Data on the location, size, and number of liver abscesses and the presence or absence of catheter drainage placement were obtained from the procedural records and image reports of the electronic medical records. Poor visual outcomes were defined as VA worse than counting fingers (CF), whereas favorable prognosis was defined by a vision of CF or better. The possible poor prognostic factors for visual outcomes were evaluated using the Pearson χ^2^ test, Fisher exact test, and Student t test for univariate analyses and binary logistic regression for multivariate analysis. All analyses were performed using SPSS (version 23.0, SPSS Inc., Chicago, IL, USA), and *p* ≤ 0.05 was considered statistically significant.
